# A Systematic Review of Risk and Protective Factors of Borderline Personality Disorder

**DOI:** 10.7759/cureus.85070

**Published:** 2025-05-30

**Authors:** Anastasia Kouklidou, Georgios Kouklidis, Vaios Dafoulis

**Affiliations:** 1 Department of Child and Adolescent Psychiatry, Ippokrateio General Hospital, Thessaloniki, GRC; 2 Department of Orthopaedics, University Hospitals Dorset NHS Foundation Trust, Dorset, GBR

**Keywords:** environmental factors in bpd, family factors in bpd, protective factors of borderline personality disorder, risk factors of bpd, social factors of bpd

## Abstract

According to the Diagnostic and Statistical Manual of Mental Disorders, Fifth Edition (DSM-5), certain criteria are used for the diagnosis of borderline personality disorder (BPD). Although there is evidence that several risk factors could predispose individuals to BPD, it is still not clear what the etiologic pathways of this personality disorder are. The aim of this systematic review has been to identify, classify, analyze and interpret data regarding risk and protective factors for BPD to inform policies and practices in terms of public health. In total, 15 studies were included in this systematic review. Ten longitudinal studies and three case-control studies analyzed individual and family risk factors for BPD. Two out of 15 were investigating possible protective factors for the disorder. Most studies were focused on family risk factors for BPD. This systematic review showed that many studies suggested possible family risk factors for the development of BPD. Evidence suggests that there are “silent” risk factors that have a long-term effect on a child’s personality and increase the risk for BPD in adult life. Following a public mental health framework, BPD prevention and promotion of mental health could contribute to an improved quality of life for all family members and better mental health status for the general population.

## Introduction and background

According to the International Classification of Diseases, Tenth Revision (ICD-10) classification of mental and behavioral disorders, borderline personality disorder (BPD) belongs to the borderline type (code F60.31) of emotionally unstable personality disorder (code F60.3) [1]. BPD is characterized by emotional instability combined with disturbed self-image, ambiguous future aims and sexual preferences, unstable relationships that may provoke exacerbation of their emotional state and extremes of idealization alternating with extremes of devaluation [2]. Suicidal threats or any action of self-harm is part of a general attempt to avoid abandonment [2]. Chronic internal emptiness, problems in controlling anger and transient paranoid ideation due to stressful conditions fulfill the diagnostic evidence for BPD, with symptoms typically emerging in early adulthood [3].

BPD prevalence ranges from 0.2% to 1.8% in the general population [4], rising to 12% in psychiatric outpatients and 22% in inpatients [5]. Its prevalence is four times more common in primary care than in the general community [6], with women comprising 75% of the general population [7]. The estimated prevalence of BPD for patients with mood disorders is 29.4%, for anxiety disorders is 21.5% and for substance use disorders is 14.7% [8]. Women with nicotine dependence have a higher prevalence 28 of developing BPD, accounting for 17.6% compared to men with nicotine dependence, whose prevalence is approximately 13.6% [8].

Evidence suggests that there is a set of risk factors associated with increased risk for BPD. Childhood sexual and physical abuse, experiences of serious domestic violence and reports of emotional and physical neglect seem to play an important role in the future development of the disorder [9]. Women with a history of early-onset abuse seem to be at greater risk for BPD [10]. Apart from traumatic experiences during childhood, genetic factors may play an important role in the disorder. According to a twin study, 221 pairs were investigated, reporting higher concordance of BPD in monozygotic rather than dizygotic twins [11]. Finally, several studies report higher rates of psychopathology in offspring of BPD mothers [12], and premature birth could be a risk factor, as it was commonly reported in BPD patients [13].

BPD affects physical health through various pathways, including its association with depression and alcohol abuse, increasing the number and the severity of suicidal attempts and, thus, reflecting a greater impact of the disorder on the mental and physical health of individuals [14]. Lifetime suicide attempt rates among individuals with BPD range from 46% to 92%, indicating a substantially higher risk, approximately 50% greater than that of the general population [14]. Self-injury and self-harm are also common in this population [15]. BPD has a great effect on the mental health status of an individual [16]. The necessity of supporting protective factors for individuals at high risk of experiencing BPD is crucial. The aim of this systematic review has been to identify, classify, analyze and interpret data regarding risk and protective factors for BPD to inform policies and practices in terms of public health.

## Review

Methods

Search Strategy and Information Sources

**Figure 1:**
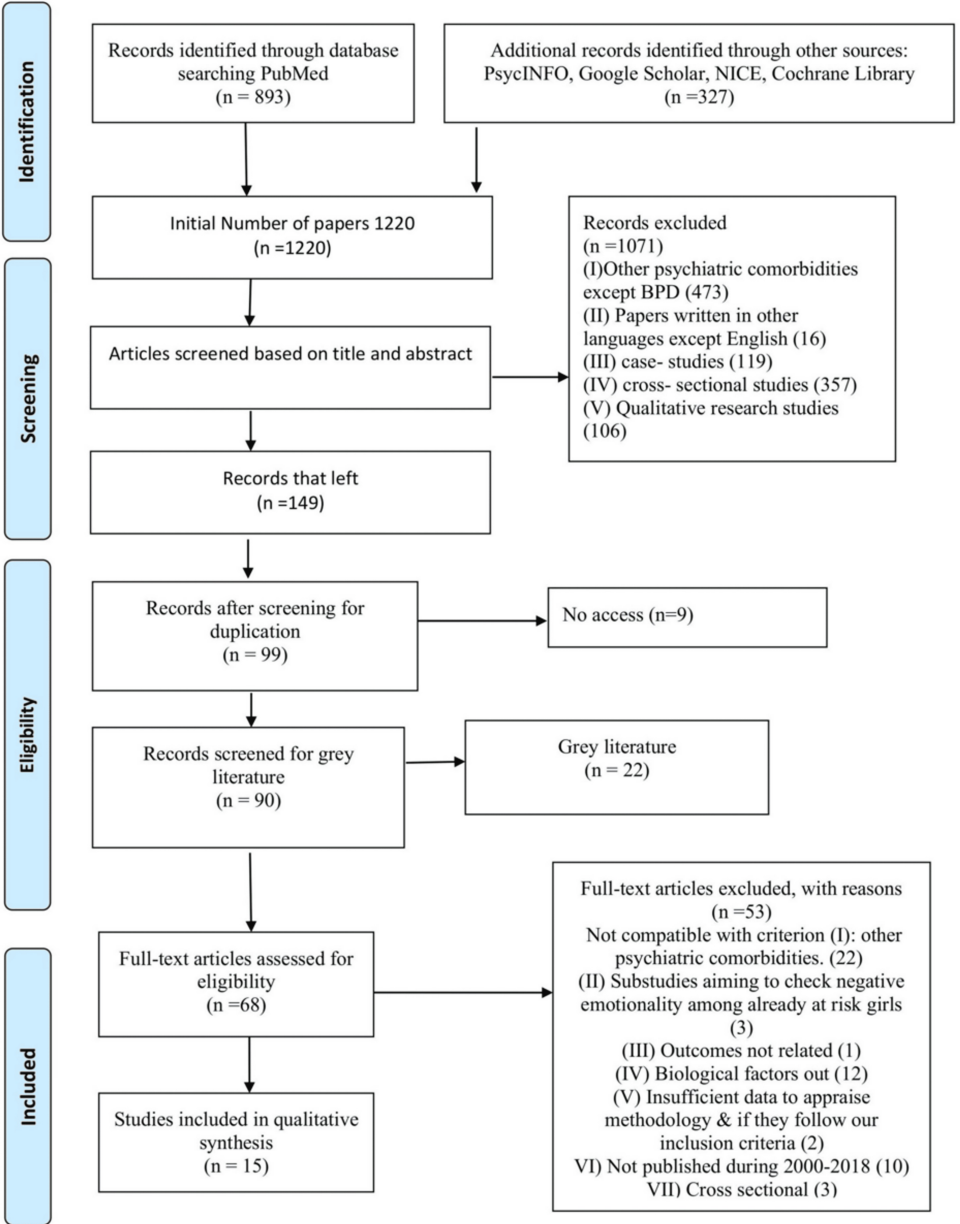
PRISMA flow chart PRISMA: Preferred Reporting Items for Systematic Reviews and Meta-Analyses

This literature review was conducted according to the Preferred Reporting Items for Systematic Reviews and Meta-Analyses (PRISMA) guidelines (Figure 1).

We searched for articles in the electronic databases of PubMed, Google Scholar, PsycINFO, NICE and Cochrane Library by using a combination of keywords. The electronic search strategy is described below:

A comprehensive literature search was conducted using the following MeSH terms and keywords: "risk factors" AND "borderline personality disorder", "protective factors" AND "borderline personality disorder", "parents" OR "parental" AND "borderline personality disorder", "family" AND "borderline personality disorder", "neurobiological factors" AND "borderline personality disorder", "environmental factors" AND "borderline personality disorder", "social factors" AND "borderline personality disorder", "cultural factors" AND "borderline personality disorder", "childhood abuse" AND "borderline personality disorder", "birth" AND "risk factors" AND "borderline personality disorder", "early traumatic life events" AND "borderline personality disorder", "attachment theory" AND "borderline personality disorder", "substance abuse" AND "borderline personality disorder", "psychological factors" AND "borderline personality disorder", "psychological trauma" AND "early childhood" AND "borderline personality disorder", "HPA axis" AND "borderline personality disorder". Search was carried out from 25/01/19 to 22/03/19. The last search was on 22/03/19.

Eligibility Criteria

For the development of the inclusion and exclusion criteria of the study, the PICO (Patient or Problem, Intervention, Comparison, and Outcome) checklist was used [17]. The key aspects according to PICO are described as follows.

Patient population or problem: Longitudinal studies and case controls, including patients diagnosed with BPD, as well as studies investigating the exposure of individuals to risk factors associated with the manifestation of BPD

Exposure: Any exposure to biological, psychological and social factors associated with the risk for or protection from the onset of BPD

Comparison or intervention (if appropriate): Not applicable

Outcome: Key determinants of BPD at biological, psychological and social levels, as well as risk and protective factors associated with the manifestation and/or etiology of BPD.

The inclusion criteria for the current systematic review are analyzed below.

Studies published between 2000 and 2018: These studies were included because BPD was not consistently recognized as an independent clinical entity prior to 2000. Earlier studies varied widely in diagnostic criteria and definitions, risking increased bias if included.

Study design: Randomized controlled trials (RCTs), longitudinal studies, cohort studies, and case-control studies

Studies specified: Studies on clinical populations diagnosed with BPD and those using the diagnostic criteria of the Diagnostic and Statistical Manual of Mental Disorders, Fifth Edition (DSM-5) or ICD-10 for BPD

Exclusion Criteria

Studies were excluded if they met the following criteria: (I) studies that were investigating other psychiatric comorbidities, except BPD; (II) papers written in other languages, except English; (III) case studies; (IV) cross-sectional studies; (V) qualitative research studies; and (VI) sources that were not formally published such as journal articles, books and presentations in conferences related to the topic. There were no criteria regarding age and sex.

Data Extraction and Quality Assessment

Study selection and data collection process: The initial number of studies gathered was 1,220, and the final number of studies selected was 15. Screening was carried out independently by two researchers by following a stepwise approach: (I) screening according to paper title, (II) screening according to abstract and (III) full-text screening process. Articles selected for final analysis met the following criteria: (I) studies published between 2000 and 2018; (II) RCTs, longitudinal studies, cohort studies, and case control studies; III) studies on clinical populations diagnosed with BPD; and IV) studies using the diagnostic criteria of DSM-5 or ICD-10 for BPD.

The selection and inclusion process was based on a set of pre-defined criteria (described in previous sections) and a consensus process implemented across studies and cases. Disagreements between the two researchers were resolved by a third researcher. Data were extracted from relevant reports by using an extraction form including variables such as publication date, population and sample, research design and key outcomes.

Risk of bias in individual and across studies: To assess the risk of bias in individual studies, the Cochrane guidelines were used. More specifically, the Cochrane Risk Of Bias In Non-Randomized Studies of Interventions (ROBINS-I) tool was used for risk of bias assessment.

Risk Assessment

The search results yielded 15 full-text articles relevant to the research question. Although no RCTs were identified, the selection included 10 longitudinal studies and five case-control studies. The longitudinal studies exhibited limitations in patient characteristics, notably a disproportionate representation of female participants compared to males. The inclusion of certain ethnicities was uneven, with more studies focusing on Caucasian populations compared to other ethnic groups. This introduces selection bias. There was a difference in the number of subjects that were recruited compared to the number of subjects that were analyzed. The major limiting factors in case-control studies were the small sample size and the development of other factors that could influence the results, such as alcohol abuse, symptoms of schizotypal personality disorder and a combination of multiple factors, which makes it difficult to isolate the etiology of the development of BPD.

**Figure 2:**
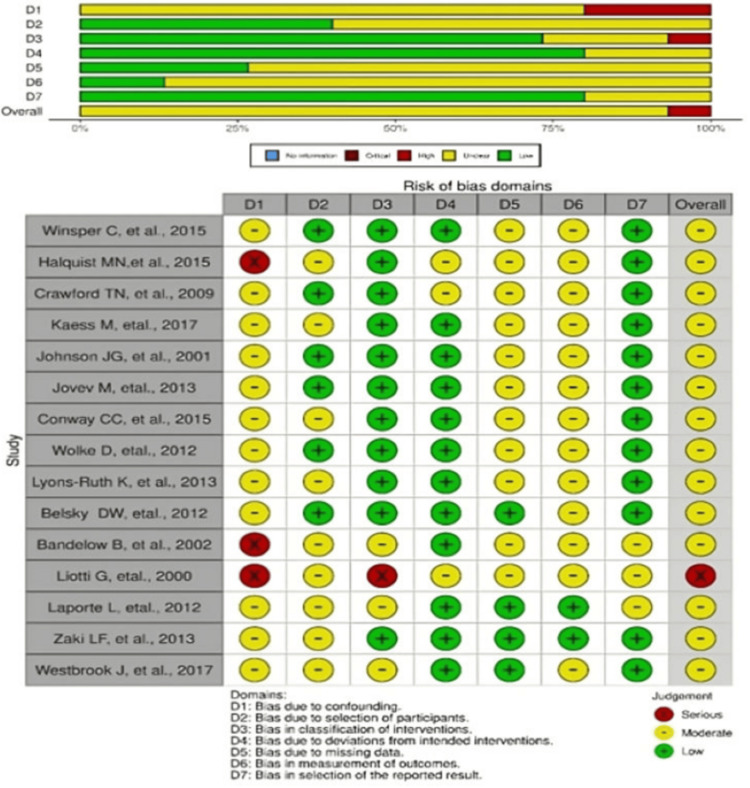
Risk of bias graph Sources: Refs. [18-32]

Regarding case-control studies, the important limiting factor was that, by design, they are retrospective studies, and hence they include a component of recall bias. It is important to consider the variability between how the interviews were conducted and the variability between time periods. There are variabilities between papers and randomization of selection of interviewers and the skills each interviewer possesses to thoroughly carry out the interview. This is a source of performance bias. These studies were not as rigorously designed as RCTs, and so there were risks of bias such as selection bias, performance bias and unclear other biases. Although the source of funding was stated by the authors, it is unclear if it has any influence on the results of the paper. Figure 2 graphically represents the quality assessment results.

The characteristics of each study and the relevant data extracted by each are described in Table 1.

**Table 1:** Data extracted from each study

Study characteristic	Data extracted
Study details & design	Reference, type of publication, study design, setting
Participants details	Inclusion & exclusion criteria, number of people recruited, number of people analyzed, age, sex
Exposure	Variables related to population exposure to the risk factors associated with BPD
Potential confounders	Variables that can affect the relationship between exposure and BPD-related outcomes
Results/Outcomes	Correlations & association between factors and/or variables indicating risk for BPD

Results

Fifteen individual studies investigating psychological, social risk and protective factors for BPD were found to meet the inclusion criteria. Specifically, 10 longitudinal studies and three case-control studies analyzed individual and family risk factors for BPD. The characteristics of these studies are illustrated in Table 2.

**Table 2:** Study characteristics and results of individual studies about psychological and social risk factors and BPD Notes: F2F Semi-structured Interview UK-CI-BPD = Face-to-Face Semi-structured Interview, PMAC-PQ = Prenatal Maternal Alcohol Consumption-Postal Questionnaires, CCEI = Crown Crisp Experimental Index, EPDS = Edinburgh Postnatal Depression Scale, FAI = Family Adversity Index, PCI = Parent Conflict Index, CTS = Conflict Tactic Scale Fulfilled, IPDE-S = International Personality Disorder Examination-Screen, CAR = Cortisol Awakening Response, CTQ = Childhood Trauma Questionnaire, PDS = Pubertal Development Scale, NYSCR = Official New York State Central Registry for Child Abuse & Neglect, DISC-I = Diagnostic Interview Schedule for Children, DPI = Disorganizing Poverty Interview, HSCL = The Hopkins Symptom Checklist, EATQ = Early Adolescent Temperament Questionnaire, K-SADS-E = The Schedule for Affective Disorders and Schizophrenia for School-Age Children—r Kiddie, SCID-II = Structured Clinical Interview for DSM-IV Axis II Personality Disorders, WISC = Wechsler Intelligence Scale for Children, SWAP-200 = Shedler-Westen Assessment Procedure, BPRCs = Borderline-Personality-Related Characteristics, DES = Dissociative Experiences Scale, Dx of BPD = Diagnosis of Borderline Personality Disorder, CIC-SR = Children in the Community Self-Report Scale, CTQ = Childhood Trauma Questionnaire, SDQ = Strengths & Difficulties Questionnaire, PDQ = Personality Diagnostic Questionnaire, DSM-IV = Diagnostic and Statistical Manual of Mental Disorders IV, Dx = Diagnosis, BPD = Borderline Personality Disorder, CPI = California Psychological Inventory

Study		Details & Design			Characteristics	Exposure/Risk factors		Results	
Winsper et al., 2015, UK [18]	Study design: Longitudinal study. Type of publication: Full text. Setting: Community sample			N: 6050 mothers & their children. Inclusion criteria: Pregnant women & children born between April 1991 & December 1992, children eligible for invitation to the 11-year annual assessment clinic. Exclusion criteria: Not answering at least eight of the nine BPD questions in UK-CI-BPD. Number of people recruited: 11,510. Number of people analyzed: 6050. Mean age of children: 11.7 years. Tools: UK-CI-BPD/DSMIV, PMAC-PQ, prenatal maternal tobacco use simple questions, CCEI, EPDS, FAI, PCI, SDQ. Sex: 48.6% males, 51.4% females. Follow-up: 11-12 years			Prenatal & postnatal maternal alcohol consumption, tobacco use, anxiety & depression. Family adversity, maladaptive parenting internalizing/externalizing symptoms. Confounding factors: Sex, birth weight	There was a significant positive association between prenatal factors with BPD. After adjustment of sex, birth weight, maternal alcohol use, smoking, anxiety & depression, after birth as well as family adversity there was a significant positive association between, internalizing & externalizing symptoms identified in children & BPD	
Halquist et al., 2015, Pittsburg [19]	Study design: Longitudinal study. Type of publication: Full text. Setting: Community: Pittsburgh Girls Study			N: 2228 girls aged 14-17 years old. Inclusion criteria: All Pittsburgh neighborhoods with 25% of their families living at or below poverty level, random sample of 50% of households in all the other neighborhoods, BPD symptoms in girls aged 14. Exclusion criteria: Not mentioned. Number of people recruited: 2450 girls aged 5-8 years old. Number of people analyzed: 2228 girls. Mean age: 14-17 years old. Sex: Girls. Tools: Separate in-house interviews for both girls & caregiver, CTS, negative emotionality subscale score, self-control subscale of the social skills ratings scale, IPDE-S, poverty was measured by whether caregivers were receiving public assistance. Follow up: 4 years			Harsh punishment in childhood. Poor self-control in childhood. Negative emotionality. Possible confounders: Race	Harsh punishment in childhood: There was a significant positive association between BPD symptoms at 14 years old with the level of harsh punishment at age 10 & the growth rate in harsh punishment between ages 10-14. Child reports of harsh punishment at age 10 revealed greater increases in poor self-control between the ages of 10 & 12 increasing the severity of BPD symptoms by the age of 14. Poor self-control in childhood: The level and the rate of growth in poor self-control in the age group of 10 & 12 years old were significant predictors of raised severity of BPD symptoms. Self-control changes among subjects in 12 & 14 years old were negatively associated with BPD symptoms. Poor self-control between ages 12 & 14 was the only important predictor of BPD symptoms change in age 14 & 17. Negative emotionality: Increased negative emotionality at age 11 was a predictor of the development of BPD symptoms in age 14	
Crawford et al., 2009, UK [20]		Study design: Longitudinal study. Type of publication: Full text. Setting: Community sample	N: 766 youth. Inclusion criteria: Participants were born over a full decade. Youths who participated in two or more follow up interviews in 1983, 1985-1986, 1991-1993, 2001-2004, random selection of family members in rural, suburban city & central city in two countries in New work. Exclusion criteria: Children outside the initial age range, families lost to follow-up, non-responders to study communications. Number of people recruited: 976 mothers with a child who was in the age group of 1 & 10 were selected randomly. Number of people analyzed: 766. Mean age: 13, 16, 22, 33. Sex: Female. Tools: PDQ, Maternal separation, CIC attachment scales, crying demanding scale, marital conflict scale, maternal inconsistency & dissatisfaction/satisfaction scale, child maltreatment form legal records, CIC. Follow-up: 2 decades				Extended early maternal separation. Possible confounders: Socioeconomic status, Temperament of the child, maternal conflict, inconsistency, dissatisfaction, child maltreatment, insecure attachment, Schizotypal personality disorders before 5 years old	Separation variables: Extended visits to relatives, mother’s withdrawal from personal/ professional goals were significant. Prolong separations from mother before the age of 5 were positively associated with BPD symptoms in adolescence & adulthood. The level of BPD symptom decline with age was less compared to those who had experienced early separations to a prolonged (Mean separation duration=15.7wks). Additional risk factors: SES was inversely related to BPD symptoms. Higher BPDS symptoms & lower rates of decline of BPD symptoms in later life showed a significant positive association with BPD symptoms. Inconsistent mothering & low maternal satisfaction were significant predictors of BPD symptoms. Parental conflict and maternal interpersonal adversities were not significant predictors of offspring BPD symptoms. Increased anxiety and avoidant attachments revealed a noteworthy increase in BPD symptoms over trajectory. Schizotypal PD symptoms did not reveal any effect on early separation and BPD symptom trajectory.	
Kaess et al., 2018, Australia [21]		Study design: Longitudinal study. Type of publication: Full text. Setting: Sample from another larger longitudinal study of adolescent development (nested cohort)	N: 69 adolescents Inclusion criteria: Subjects who participated in all 3 assessments at T1-T3. Exclusion criteria: Subjects receiving medication with an established effect on hormone levels, oral sores, failure to comply with collection protocols & cortisol outliers. Number of people recruited: 2479. Number of people analyzed: 69. Mean age: 15 years old. Sex: 30 females, 39 males. Tools: CAR, CTQ, PDS, salivary samples, Elecsys E immuno-analyzer, BPD Subscale of Adolescent Personality Disorder. Follow-up: Not mentioned				Childhood maltreatment. Confounding factors: Pubertal development, Sex.	Maltreatment, BPD Symptoms & Cortisol Awakening Response (CAR): No significant effect of BPD symptoms compared to childhood maltreatment on cortisol awakening response. Maltreatment predicted a lower cortisol awakening response. Sex served as a moderator. Childhood maltreatment & BPD symptoms predicted CAR in females but no in males. Early BPD symptoms serve as moderator in the effect of childhood maltreatment on HPA axis reactivity in later life. Childhood maltreatment has no association with lower CAR when BPD symptoms were also present.	
Johnson et al., 2001, Columbia [22]		Study design: Longitudinal study. Type of publication: Full text: Setting: Community sample	N: 793 mothers & children. Inclusion criteria: Randomly sampled form two counties in upstate New York interviewed in 1975 and at least two times in 1983, 1985-1986 and 1991-1993. Exclusion criteria: Not mentioned. Number of people recruited: 976. Number of people analyzed: 793. Mean age: 5, 14, 16, 22. Sex: 390 females, 403 males. Tools: Maternal & psychosocial interviews, NYSCR, DISC-I, PDQ, DPI, DSM-IV, CPI, HSCL. Follow-up: Not mentioned				Childhood verbal abuse. Confounding factors: Offspring temperament, childhood physical abuse, sexual abuse, neglect, physical punishment during childhood, parental education, parental psychopathology, existence of other psychiatric disorders, sex	Offspring with experiences of maternal verbal abuse during childhood were three times more likely to develop borderline, narcissistic, obsessive-compulsive & paranoid PDs during adolescence or early adulthood. Childhood verbal abuse: No interaction with childhood physical & sexual abuse or neglect to predict PD symptoms during early adolescence and adulthood.	
Jovev et al., 2013, Australia [23]		Study design: Longitudinal study. Type of publication: Full text. Setting: Sample form another large study	N: 205 children aged 11 & 13 years. Inclusion criteria: Children randomly selected from government, independent & Catholic primary schools in metropolitan Melbourne, aged 10-12, screened with EATQ. Exclusion criteria: Not mentioned. Number of people recruited: 245. Number of people analyzed: 205. Mean age: 11 and 13 years. Sex: 121 out of 245 males. Tools: EATQ, CIC-SR, CTQ. Follow-up: 2 years				Abuse and neglect. Confounding factors: Type of schools, SES, parental education, ethnicity, decrease number of reporting sexual abuse	Abuse, neglect & BPD: There is a significant positive association between high neglect scores and the high number of BPD symptoms. Abuse seemed to moderate the relationship between temperament & BPD symptoms. Temperament, abuse, neglect & BPD: There was a significant negative association between abuse and affiliation in adolescence with high BPD symptoms. Temperament during early adolescence and parental maltreatment are involved in the prediction of BPD symptoms. Neglect and abuse were independent predictors of high BPD symptoms. Temperament was moderating the effect of parental abuse & neglect	
Conway et al., 2015, Los Angeles [24]		Study design: Longitudinal study. Type of publication: Full text. Setting: Community sample	N: 700. Inclusion criteria: Youths aged 15 years, Mater-University Study of Pregnancy in Brisbane, fulfilled BPD assessments. Exclusion criteria: Not mentioned. Number of people recruited: 815. Number of people analyzed: 700. Mean age: 15. Sex: 362 females. Tools: K-SADS-E, SCID-II, SCID-Q, UCLA, life stress interview. Follow-up: 5 years				Acute stress occurring over the past year & chronic stress over 6 months Maternal externalizing disorder history Offspring internalizing disorder history. Family stressors. School-related stressors. Confounding factors: Sex (women reported 30% more BPD symptoms than men), Family income, parental education level	Only maternal externalizing disorder, offspring internalizing disorder, school stressors and family stressors had a significant positive association with BPD symptoms. Adolescent internalizing history, acute stress & BPD: Acute stress exposure-predicted BPD symptoms had a significant effect on subjects that weren’t diagnosed with internalizing disorders. Maternal externalizing history & BPD: Maternal externalizing disorders showed the strongest effect among other risk factors that could contribute to offspring BPD features. School-related stressors & BPD: Stressful conditions at school, attendance problems indicated high risk of BPD features	
Wolke et al., 2012, USA [25]		Study design: Prospective, longitudinal observation study. Type of publication: Full text. Setting: Community sample	N: 6050 mothers and their children. Inclusion criteria: Children from the southwest of England who had expected birth date between 1 April 1991 & 31 December 1992. Exclusion criteria: Not mentioned. Number of people recruited: 10,000. Number of people analyzed: 6050. Mean age: 12 years. Sex: Not mentioned. Tools: DSM-IV, F2F interviews, SDQ, parental, child, teacher reports, FAI, WISC. Follow-up: 12 years				Bullying during childhood. Confounding factors: Family adversity, parental behaviors, sexual abuse, presence of axis I diagnosis, IQ, birth weight, sex	Bullying during childhood: Individuals who reported bullying (chronic or not) or combination of relational & apparent victimization by their peers had high risk of developing BPD symptoms	
Lyons-Ruth et al., 2013, USA [26]		Study design: Longitudinal study. Type of publication: Full text. Setting: Community sample	N: Adolescents from 56 low income families. Inclusion criteria: Not mentioned. Exclusion criteria: Not mentioned. Number of people recruited: 76. Number of people analyzed: 56. Mean age: 19.9 years. Sex: Not mentioned. Tools: SCID-II, self-report measures, videotape, interviews. Follow-up: 21 years				Maltreatment, suicide. attachment. Confounding factors: Ethnicity, SES, age, infant/childhood outcome assessments, clinical status, gender	BPD symptoms, suicidal behavior and abuse: 53% revealed constant suicidal threats/self-injury, 89% exhibited one of these symptoms. There was a significant positive association between the severity of abuse with the severity BPD symptoms. Attachment in infancy: Maternal withdrawal in infancy was the most important predictor of future development of BPD symptoms. Early maternal withdrawal was predicting future BPD symptoms independently of any identification of disorganized-controlling behavior during childhood. Child attachment: Disorganized-controlling behavior at the age of 8 was significant predictor of the severity of BPD symptoms at the age of 20	
Belsky et al., 2012, UK [27]		Study design: Longitudinal study. Type of publication: Full text. Setting: Members of a longitudinal twin study	N: 1116 pairs of same sex twins (54% monozygotic). Inclusion criteria: British children, born in England & Wales in 1994-1995, same sex, 5 years old. Exclusion criteria: Νot mentioned. Number of people recruited: 2232. Number of people analyzed: Not mentioned. Mean age: Not mentioned. Sex: 51% of females. Tools: Home visit evaluations, interviews with mothers, DSM-IV, SWAP-200-A, children’s report, BPRCs. Follow-up: 12 years				Family history of psychiatric illness. Harsh treatment. Confounding factors: Twin concordance	Harsh treatment (physical maltreatment & maternal negative expression of emotion) before the age of 10 predicted BPRCs at 12 years old. Children reporting higher maternal negative expressed emotion showed an increased number of BPRCS. Environmental mediation was a stronger risk factor in children with family history of psychiatric illness.	
Bandelow et al., 2003, Germany [28]		Study design: Case-control study. Type of publication: Full text. Setting: Outpatient setting	N: 66 patients with BPD. N: 109 controls. Inclusion criteria: Not mentioned. Exclusion criteria: Not mentioned. Number of people recruited: 155 controls. Number of people analyzed: 66 patients. Mean age of patients: 30. Mean age of controls: 32. Sex: 71.2% women, 60.6% control. Tools: DSM-IV, reports from patients. Follow-up: Not mentioned				Early traumatic life events. Parental attitudes. Family history: Confounding factors: Unknown relationships between the factors	Traumatic childhood events: No statistical differences between the groups. Childhood illness: Patient group reported a major disease in childhood with higher duration as opposed to the control group. Physical handicaps: Patient group reported an increased number of handicapped siblings compared to the control group. Social environment: Unemployment of mother was higher in-patient group. Violence in families: Highly reported in patient group. Fathers were highly reported to abuse physically their children. Sexual abuse: Childhood sexual abuse with or without penetration was significantly more frequent in-patient groups. Parental attitude & rearing styles: Patient groups reported less affection, care and more inappropriate rearing styles. Psychiatric disorders in the family: BPD group reported a broad spectrum of psychiatric morbidity in their first-degree relatives	
Liotti et al., 2000, UK [29]		Study design: Case-control study Type of publication: Full text. Setting: Hospital	N: 66 cases, N: 146 controls. Inclusion criteria: Dx of BPD. Exclusion criteria: Number of people recruited: 66. Number of people analyzed: 66. Mean age: Not mentioned. Sex: Males & females Tools: DSM III, SCID II, DES, Questionnaire on Loss Events, Infancy Trauma Interview				Early traumatic experiences & losses. Confounding factors: Age, size of family, patient’s education, mother’s education, type of patient (in-patient or outpatient)	Early traumatic experiences & serious losses of the mother: Patient’s traumatic experiences during early life as well as losses experienced by patient’s mother within two years after birth were characterized as independent predictors of BPD development	
Laporte et al., 2012, Canada [30]		Study design: Case-control study. Type of publication: Full text. Setting: Psychiatric clinics	N: 53 patients diagnosed with BPD were compared to 53 healthy sisters. Inclusion criteria: Dx of BPD, sister closest in age, age difference within 7 years of each other, full siblings living with at least one shared biological parent during their early life. Exclusion criteria: Number of people recruited: 88. Number of people analyzed: 53. Age: 18-45 years old. Sex: Not mentioned. Tools: DSM-IV, SCID, childhood trauma interview, parental bonding instrument				Childhood abuse & neglect. Dysfunctional parent-child relations peer victimization. Confounding factors: Temperament factors, demographic characteristics	Childhood abuse & neglect: Both BPD groups and healthy siblings had similar rates of emotional abuse as well as physical neglect and abuse. Significant psychosocial impairment affects BPD patients at a higher level in terms of work, relationship and risk of substance abuse. Parent-child relations: Maternal care & overprotection were less likely to be reported by BPD group. Peer victimization: Peer delinquency revealed higher probability of being reported by BPD subjects. BPD group had lower odds	

Two out of 15 were investigating possible protective factors for the disorder. The characteristics of these studies are illustrated in Table 3.

**Table 3:** Psychological factors that have a protective role against BPD Note: NSSI = Non-suicidal Self-Injury, PDs = Personality Disorders, SIDP-IV = Semi-structured Interview Designed to Assess the Presence of Axis II Personality Disorders, ISAS = Inventory of Statements About Self-Injury, SIDP-I = Structured Interview for DSM-IV Personality Disorders, Version I, RRS = Rumination Responses Scale, SNAP-2 = Subscale from the Schedule for Nonadaptive & Adaptive Personality-2, TMMS = Trait Meta Mood Scale, BPD group = Borderline Personality Disorder Group, DSM-IV = Diagnostic and Statistical Manual of Mental Disorders IV, Dx = Diagnosis, Hx = History

Study	Details & Design	Characteristics	Protective factors	Results
Zaki et al., 2013, Columbia [31]	Study design: Case-control. Type of publication: Full text. Setting: From a larger study	N: 38 BPD participants. N: 42 healthy subjects. Inclusion criteria: Dx of BPD according to DSM-IV, hx of NSSI. Exclusion criteria for BPD group: Evidence of primary psychotic disorder, current substance abuse/withdrawal, cognitive impairment and illiteracy. Exclusion criteria for healthy group: Met more than 2 criteria for any PD or more than 10 criteria through all PDs, with current or partially remission of axis I disorders in the year before the interview date, received psychiatric treatment or had SCID-I Global Assessment of Functioning scores <80, any history of self-injurious behavior. Number of people recruited: 81. Number of people analyzed. Age: 29.89 (BPD group), 32.50 (healthy group). Sex: 84% female (BPD group), 83% (healthy). Tools: DSM-IV, SIDP-IV, SIDP-I, ISAS, RRS, 21-day computerized experience-sampling diary	Emotion differentiation. Confounding factors: Ethnicity, currently on psychiatric medication, age, gender, education	There is a significant positive association between rumination and NSSI under the effect of high and moderate negative emotion differentiation in the BPD group
Westbrook et al., 2016, USA [32]	Study design: Case-control. Type of publication: Full text. Setting: From a larger study	N: 293. Inclusion criteria: Not mentioned. Exclusion criteria: Not mentioned. Number of people recruited: Not mentioned. Number of people analyzed: 293. Sex: 53.9% female. Mean age: 43.1. Tools: SNAP-2, semi-structured interviews, self-report of childhood physical abuse, childhood trauma interview, TMMS, 10-item negative effect scale	Emotional awareness. Confounding factors: Education level, ethnicity, marital status	Negative emotion differentiation had a protective role in BPD patients with episodes of NSSI

Most studies were focused on family risk factors for BPD. Prenatal adversities, childhood abuse and neglect, childhood verbal abuse, dysfunctional parent-child relations, early traumatic experiences and losses, parental attitudes, family history of psychiatric illness, peer victimization, harsh punishment and bullying during childhood, acute and chronic stress, maternal externalizing disorder history and school stressors were analyzed. Individual factors such as poor self-control in childhood, negative emotionality and offspring internalizing disorder were also investigated.

Family factors are the most investigated factors among these studies and seem to play a key role in the manifestation of the disorder. Winsper et al. found prenatal adversities were significantly associated with BPD at 11-12 years old, and there was a significant association between internalizing and externalizing symptoms identified in children with BPD [18]. Hallquist et al. reported that BPD symptoms at 14 years old were significantly associated with the level of harsh punishment at age 10 and the growth rate in harsh punishment between ages 10 and 14 [19]. The level and rate of growth in poor self-control in the age group of 10-12 years old were significant predictors of raised severity of BPD symptoms [19]. Increased negative emotionality at age 11 was a predictor of the development of BPD symptoms at the age of 14. Socioeconomic status (SES) was inversely related to BPD symptoms [19]. Crawford et al. suggested that prolonged separation from mother before the age of five was associated with BPD symptoms in adolescence and adulthood [20].

Kaess et al. investigated childhood maltreatment in four longitudinal studies and two case-control studies [21]. Childhood maltreatment predicted a lower cortisol awakening response (CAR). Sex moderated this effect, with a negative association in the female group and a positive association in the male group. Childhood maltreatment and BPD symptoms predicted CAR in females but not in males. Jovev et al. reported that neglect and abuse were independent predictors of high BPD symptoms [23]. Johnson et al. found that offspring who experienced maternal verbal abuse during childhood were three times more likely to develop BPD during adolescence or early adulthood compared to those who did not report any verbal abuse at that period of their life [22]. Laporte et al. identified significant psychosocial differences between the groups, affecting BPD patients at a higher level in terms of work, relationship and risk of substance abuse [30]. Bandelow et al. identified that childhood sexual abuse, with or without penetration, was significantly more frequent in the BPD group [28]. In the end, Belsky et al. suggested that harsh treatment (physical maltreatment and maternal negative expression of emotion) before the age of 10 predicted borderline personality-related characteristics (BPRCs) at 12 years old [27].

Parental behavior, family, school and social stressors were tested in six longitudinal studies and two case-control studies. Crawford et al. identified inconsistent mothering and low maternal satisfaction as significant predictors of BPD symptoms, with anxiety and avoidant attachment increasing symptom over trajectory [20]. Conway et al. demonstrated that maternal externalizing disorders showed the strongest effect among other risk factors that could contribute to offspring BPD features [24]. In consonance with Lyons-Ruth et al., who reported that maternal withdrawal in infancy was the most important predictor of future BPD symptoms [26]. In a case-control study, Bandelow et al. showed that BPD reported less affection, care and more inappropriate rearing style as opposed to the control group, higher maternal unemployment and greater paternal physical abuse [28]. Another case-control study of Liotti et al. revealed that patients’ traumatic experiences during early life, as well as losses experienced by patients’ mothers within two years after birth, were independent predictors of the development of BPD [29]. Maternal care and overprotection were less likely to be reported by the BPD group as well. In a longitudinal study of Wolke et al., individuals who reported bullying by their peers had a high risk of developing BPD symptoms [25].

Conway et al. reported that stressful conditions at school, such as difficulties in interpersonal relationships with other students, teachers and school absence, indicated a high risk of BPD features [24]. Belsky et al. showed that environmental mediation was the strongest risk factor in children with a family history of psychiatric illness [27]. Similarly, a case-control study by Bandelow et al. identified a broad spectrum of psychiatric morbidity in their first-degree relatives of the BPD group [28].

Individual factors were also tested by three longitudinal studies. Jovev et al. indicated that temperament during early adolescence and parental maltreatment are involved in the prediction of BPD symptoms [23]. In agreement with Conway et al., temperament was moderating the effect of parental abuse and neglect [24]. In case of adolescent internalizing disorder history, acute stress exposure predicted BPD symptoms as opposed to externalizing disorder history, which showed no interaction with acute stress [24]. Acute stress had a significant effect on subjects who were not diagnosed with internalizing disorders compared to those who received the diagnosis [24]. Lyons-Ruth et al. reported that the severity of abuse was significantly associated with the severity of BPD symptoms [26]. Disorganized-controlling behavior at the age of eight was a significant predictor of the severity of BPD symptoms at the age of 20 [26].

Emotional awareness was identified as a protective factor against BPD in two case-control studies. Zaki et al. confirmed a significant positive association between rumination and non-suicidal self-injury (NSSI) under the effect of high and moderate negative emotion differentiation was observed in the BPD group [31]. Westbrook et al. suggested that negative emotion differentiation had a protective role in BPD patients with episodes of NSSI [32]. Emotional awareness has a protective role in BPD symptoms by moderating the effects of childhood abuse in terms of behavioral dysfunction and inability to relate.

Discussion

Summary of Evidence

This study was conducted to identify all the possible determinants that could increase the risk of developing BPD by following the M. Barry model of mental health promotion and disease prevention. Protective factors that could prevent or decrease the risk of BPD symptoms were also included in this systematic review. After searching in several databases, we found 15 papers following our inclusion criteria that investigated the psychological and social factors for BPD and the protective factors for developing this disorder. Ten longitudinal studies [18-27] and three case-control studies [28-30] mainly investigated family risk factors for BPD. Four out of 10 longitudinal studies [19,20,23,24] also analyzed individual risk factors, such as poor self-control in childhood, negative emotionality and offspring internalizing disorder. Two out of 15 were investigating possible protective factors for the disorder [31,32]. Ten longitudinal studies were analyzing familiar factors, such as prenatal adversities and postnatal factors, extended early maternal separation, harsh punishment, physical, sexual abuse, neglect, childhood verbal abuse, SES, inconsistent mothering and low maternal satisfaction, history of maternal externalizing disorder, family stressors, school-related stressors, bullying during childhood and family history of psychiatric illnesses. Emotional awareness was investigated by two case-control studies as a possible protective factor against BPD [31,32].

Influence of Biological Factors in BPD

Although we focused on psychological and social factors for BPD, it is important to consider studies that investigated several biological factors for the development of BPD. Hammen et al. found that the oxytocin receptor gene (OXTR rs53576) significantly moderates future BPD symptoms, after considering family background, with homozygote subjects exhibiting fewer BPD features regardless of family background [33]. Similarly, Amad et al. reported significant associations between FKBP5 polymorphisms and BPD, among them rs9470080 and rs3800373 associated with physical (p=0.01) and emotional (p=0.03) abuse, respectively [34]. It is important to clarify that not all these interactions were statistically significant after many testing corrections.

Genetic factors were further tested in a twin study by Reichborn-Kjennerud et al. found that one heritable BPD factor influenced all nine BPD criteria and was strongly affected by genetic factors with a heritability of 55% and by environmental factors (45%) [35]. Additionally, Martin-Blanco et al. reported increased methylation of NR3C1, which is the promoter area of the glucocorticoid receptor gene, revealing a positive correlation with physical abuse and emotional neglect [36]. Higher NR3C1 methylation was associated with self-injurious behavior and prior hospitalizations, suggesting a possible predisposition to increased clinical severity.

Comparison with Prior Systematic Reviews

Although there are some efforts in the field, the etiopathology of BPD remains an under-investigated issue. Only three systematic reviews exploring the factors associated with the development and manifestation of BPD were identified in the literature.

Family factors and individual factors that were identified in this systematic review were also highlighted in a systematic review of Stepp et al., in which only longitudinal studies were included [37]. In contrast to our criteria, that review included papers published through 2015, and there was no exclusion criterion for psychiatric comorbidity. Researchers found that low SES, stressful life conditions, maternal psychopathology, poor parental skills, childhood maltreatment including physical, sexual abuse and neglect were the strongest family factors for developing BPD and among individual factors: low IQ levels, increased levels of negative affect, impulsivity and internalizing and externalizing disorders were the strongest individual factors for BPD.

Our systematic review aligns with prior research, confirming key factors in BPD etiology. Low SES inversely related to BPD symptoms, while symptoms of BPD at 14 years old were significantly associated with the level of harsh punishment at the age of 10, as well as the harsh punishment with the developmental rate between the ages of 10-14 [19]. Another common finding was that increased negative emotionality at age 11 and poor self-control at ages 10 and 12 years old were significant predictors of the development and severity of BPD symptoms, respectively [20]. Prolonged separation from mother before the age of five was associated with BPD symptom manifestation during adolescence and adulthood [20]. Finally, prenatal adversities, family history of psychiatric illness, parental behavior, maternal withdrawal in infancy and family, school and social stressors were identified in both systematic review studies [18,20,26].

Another systematic review that can be compared with the present study was that of Ibrahim et al. [38]. They were focused on the investigation of maltreatment and its association with the development of borderline features during childhood. In contrast to our inclusion criteria, this review included longitudinal and cohort, case-control and cross-sectional studies. The lack of individual studies investigating the etiopathology of BPD led most researchers to include all kinds of research design studies, even those providing weak evidence and susceptible to high risk of bias. It was found that there was a significant association between maltreatment and adult BPD/borderline features during childhood, suggesting that maltreatment was a significant risk indicator for borderline features in childhood. These findings were aligned with our results, identified in four longitudinal and two case-control studies.

de Aquino Ferreira et al. identified childhood sexual abuse as an important risk factor for developing BPD, reporting a higher number of reported adult sexual abuse in BPD subjects as opposed to other personality disorders [39]. Their review included studies in English and Portuguese (1997-2017), and the time of published studies involved in their review was broader (January 1997 until January 2017) compared to ours, which focused solely on English studies (2000-2018). According to the literature, extreme adverse events such as physical and sexual abuse have been thoroughly described as strong risk factors for BPD. Additionally, it has been observed that risk factors such as inconsistent parenting, low maternal satisfaction, increased anxiety and avoidant attachments were significant predictors of BPD symptoms.

Strengths and Limitations

Among the strengths of this systematic review are the strict inclusion criteria used to identify robust studies assessing the risk of developing BPD. For this reason, all the studies recruiting subjects who were diagnosed with other psychiatric diseases were excluded. The present study was carried out according to the PRISMA guidelines. A thorough search was initially conducted to identify all the possible risk factors with biological, psychological and social origin and structural, community and individual risk factors. In this systematic review, only longitudinal and case-control studies were included, whereas cross-sectional studies, case studies and qualitative studies were excluded. This choice was made due to the high risk of bias in terms of causal relationships between the manifestation of BPD and the related risk factors. Our aim has been to create a strong evidence base to provide accurate, credible and high-quality information.

However, there are several limitations in this review. The studies included were written in the English language and primarily involved Western populations, which may affect the generalizability of the findings. We recommend that future research include studies from a broader range of cultural and geographic contexts to enhance the applicability of results globally. Another limitation was that we had no possibility of including RCTs, which are more reliable for the justification of cause-and-effect relationships. This review's most recent included study was published in 2019. As the study selection was restricted at that time, more recent research published since then was not included.

Implications and Future Directions

The findings of this review underline the importance of early identification and intervention targeting crucial risk factors such as childhood maltreatment, family dysfunction, and socioeconomic challenges to aid in the prevention of the development of BPD. Although current evidence focuses on risk factors, there is a notable gap in the exploration of protective factors. Future studies should prioritise greater diversity in sample populations, employ rigorous longitudinal and experimental designs and integrate biological, psychological and social factors to better understand BPD’s complex etiology and improve current prevention and treatment approaches.

## Conclusions

This systematic review showed that most studies suggested possible family risk factors for the development of BPD. Individual factors such as poor self-control in childhood, negative emotionality and offspring internalizing disorder were also investigated. Since most studies analysed in this review were longitudinal studies, the reliability of the results was significant. Notwithstanding, the need to conduct high-quality studies that address with greater certainty the etiology of BPD is pivotal, considering the increased suicidal rate of BPD.
